# How can automated linguistic analysis help to discern functional cognitive disorder from healthy controls and mild cognitive impairment?

**DOI:** 10.1192/bjo.2021.78

**Published:** 2021-06-18

**Authors:** Lizzie Beavis, Ronan O'Malley, Bahman Mirheidari, Heidi Christensen, Daniel Blackburn

**Affiliations:** 1 The University of Sheffield; 2 Department of Neuroscience, University of Sheffield, Sheffield Teaching Hospitals NHS Foundation Trust, Department of Neurology; 3 Dept of Computer Science, University of Sheffield; Heidi Christensen, Dept of Computer Science, University of Sheffield

## Abstract

**Aims:**

The disease burden of cognitive impairment is significant and increasing. The aetiology of cognitive impairment can be structural, such as in mild cognitive impairment (MCI) due to early Alzheimer's disease (AD), or in functional cognitive disorder (FCD), where there is no structural pathology. Many people with FCD receive a delayed diagnosis following invasive or costly investigations. Accurate, timely diagnosis improves outcomes across all patients with cognitive impairment. Research suggests that analysis of linguistic features of speech may provide a non-invasive diagnostic tool. This study aimed to investigate the linguistic differences in conversations between people with early signs of cognitive impairment with and without structural pathology, with a view to developing a screening tool using linguistic analysis of conversations.

**Method:**

In this explorative, cross-sectional study, we recruited 25 people with MCI considered likely due to AD, (diagnosed according to Petersen's criteria and referred to as PwMCI), 25 healthy controls (HCs) and 15 people with FCD (PwFCD). Participants’ responses to a standard questionnaire asked by an interactional virtual agent (Digital Doctor) were quantified using previously identified parameters. This paper presents statistical analyses of the responses and a discussion of the results.

**Result:**

PwMCI produced fewer words than PwFCD and HCs. The ratio of pauses to speech was generally lower for PwMCI and PwFCD than for HCs. PwMCI showed a greater pause to speech ratio for recent questions (such as ‘what did you do at the weekend?’) compared with the HCs. Those with FCD showed the greatest pause to speech ratio in remote memory questions (such as ‘what was your first job?’). The average age of acquisition of answers for verbal fluency questions was lower in the MCI group than HCs.

**Conclusion:**

The results and qualitative observations support the relative preservation of remote memory compared to recent memory in MCI due to AD and decreased spontaneous elaboration in MCI compared with healthy controls and patients with FCD. Word count, age of acquisition and pause to speech ratio could form part of a diagnostic toolkit in identifying those with structural and functional causes of cognitive impairment. Further investigation is required using a large sample.

